# Exploring microRNA Signatures of DNA Damage Response Using an Innovative System of Genotoxic Stress in *Medicago truncatula* Seedlings

**DOI:** 10.3389/fpls.2021.645323

**Published:** 2021-03-09

**Authors:** Carla Gualtieri, Maraeva Gianella, Andrea Pagano, Tiziano Cadeddu, Susana Araújo, Alma Balestrazzi, Anca Macovei

**Affiliations:** ^1^Plant Biotechnology Laboratory, Department of Biology and Biotechnology “L. Spallanzani”, University of Pavia, Pavia, Italy; ^2^Instituto de Tecnologia Química e Biológica António Xavier, Universidade Nova de Lisboa, Oeiras, Portugal; ^3^Association BLC3, Technology and Innovation Campus, Centre BIO- R&D Unit, Lagares da Beira, Portugal

**Keywords:** DNA damage response, microRNA, genotoxicity, camptothecin, NSC120686, tyrosyl-DNA phosphodiesterase 1, seedling development

## Abstract

One of the challenges that living organisms face is to promptly respond to genotoxic stress to avoid DNA damage. To this purpose, all organisms, including plants, developed complex DNA damage response (DDR) mechanisms. These mechanisms are highly conserved among organisms and need to be finely regulated. In this scenario, microRNAs (miRNAs) are emerging as active players, thus attracting the attention of the research community. The involvement of miRNAs in DDR has been investigated prominently in human cells whereas studies in plants are still scarce. To experimentally investigate the involvement of plant miRNAs in the regulation of DDR-associated pathways, an *ad hoc* system was developed, using the model legume *Medicago truncatula*. Specific treatments with camptothecin (CPT) and/or NSC120686 (NSC), targeting distinct components of DDR, namely topoisomerase I (TopI) and tyrosyl-DNA phosphodiesterase 1 (TDP1), were used. Phenotypic (germination percentage and speed, seedling growth) and molecular (cell death, DNA damage, and gene expression profiles) analyses demonstrated that the imposed treatments impact DDR. Our results show that these treatments do not influence the germination process but rather inhibit seedling development, causing an increase in cell death and accumulation of DNA damage. Moreover, treatment-specific changes in the expression of suppressor of gamma response 1 (*SOG1*), master-regulator of plant DDR, were observed. Additionally, the expression of multiple genes playing important roles in different DNA repair pathways and cell cycle regulation were differentially expressed in a treatment-specific manner. Subsequently, specific miRNAs identified from our previous bioinformatics approaches as putatively targeting genes involved in DDR processes were investigated alongside their targets. The obtained results indicate that under most conditions when a miRNA is upregulated the corresponding candidate target gene is downregulated, providing an indirect evidence of miRNAs action over these targets. Hence, the present study extends the present knowledge on the information available regarding the roles played by miRNAs in the post-transcriptional regulation of DDR in plants.

## Introduction

During their lifespan, plants continuously face stressful conditions (caused by exogenous and endogenous factors) that affect plant growth and development. Considering their sessile lifestyle, plants are provided with incredible genomic plasticity. For instance, the metaphorical “perceptron,” defined as an information-processing system composed of several processing units with biochemical connections, enables the selection of the most suitable options for coping with the changing environment (Scheres and Van der Putten, 2017). Linked to this, DNA damage response (DDR) is among the main strategies used by plant cells to safeguard their genome and therefore plant growth and development. To briefly define it, DDR is an intricated signal transduction network involving many players that act as DNA damage sensors, signal transducers, mediators, and effectors, working together to coordinate appropriate responses depending on the type of stimuli. A recent bibliometric study illustrates that DDR is generally far less studied in plants as compared to mammals but the interest in plant DDR research is expanding in view of future agricultural applications (Gimenez and Manzano-Agugliaro, 2017). Coincidently, it is also opportune to pinpoint that DDR is an evolutionarily conserved pathway in eukaryotes, although kingdom-specific variations are encountered (see reviews by Yoshiyama et al., 2013a; Nikitaki et al., 2018; Nisa et al., 2019). Just to cite some differences, suppressor of gamma response 1 (SOG1) is the proposed master-regulator of plant DDR, acting as a functional homolog of the mammalian p53 (Yoshiyama et al., 2009, 2013a,b). As a transcription activator, SOG1 regulates the expression of DNA repair- and cell cycle-related genes (Bourbousse et al., 2018; Ogita et al., 2018). Besides, SOG1-independent pathways have been also proposed to work in plant DDR; though the molecular mechanism is not yet fully understood, it is believed that these may include the E2F-RBR1 (RetinoBlastoma Related 1) complex, comprising transcription regulators that control the entry in the S-phase of the cell cycle (Berckmans and De Veylder, 2009). The E2Fa transcription factor also participates in DNA replication and DNA damage repair (Roa et al., 2009; Gutzat et al., 2012).

Ultimately, DDR enables the activation of cell cycle checkpoints as well as specific DNA repair mechanisms (Hu et al., 2016). Hence, the recognition and repair of DNA damage involve both the activation of DNA repair processes as well as the regulation of the cell cycle arrest, allowing the necessary time for DNA lesions to be corrected prior to cell cycle initiation. If DNA repair processes are impaired, changes in the cell cycle, transcription, and protein synthesis are encountered as well (Britt, 1999; Bray and West, 2005). Among the DNA damage repair mechanisms, some are highly specialized for specific types of damage whereas others work in a more generalized manner. It is also important to recognize that different DNA repair pathways have overlapping functions and can share key enzymes. For instance, Tyrosyl-DNA-phosphodiesterase 1 (TDP1), involved in the removal of topoisomerase I (TopI)-DNA covalent complexes (Yang et al., 1996; Pouliot et al., 1999), has been associated with both base excision repair (BER; Lebedeva et al., 2011; Donà et al., 2013) and DNA-protein crosslink (DPC) repair (Enderle et al., 2019a,b). Studies in model plants like *Arabidopsis thaliana* and *Medicago truncatula* showed that the lack of TDP1 function led to the development of dwarf genotypes sensitive to DNA damage with impaired DNA repair and cell cycle activities (Lee et al., 2010; Kim et al., 2012; Donà et al., 2013; Sabatini et al., 2016). The presence of a small subfamily of *TDP1* genes (composed of *TDP1α* and *TDP1β*) was highlighted in plants and it has been shown that the two genes do not have overlapping functions and they are differentially expressed in a species-, tissue-, and stress-specific manner (Macovei et al., 2010; Donà et al., 2013; Sabatini et al., 2017; Mutti et al., 2020).

To take place properly, the DDR system requires advanced regulatory mechanisms, which are not yet fully understood. In this context, microRNAs (miRNAs), a class of small, non-coding RNAs (~21–22 nt) that play key regulatory roles in gene expression at a post-transcriptional level (Bartel, 2004), may participate in the regulation of DDR. This aspect is quite recent and insufficiently explored, especially within the plant kingdom. Studies in human cells demonstrated that miRNAs are involved in the regulation of different components of the DDR machinery (Zhang and Peng, 2015). For instance, miR-24, miR-138, miR-182, miR-101, miR-421, miR-125b, and miR-504 were identified as crucial regulators of H2AX, BRCA1, ATM, or p53. Other such examples include miR-96, miR-155, miR-506, miR-124, miR-526, and miR-622b, shown to be involved in homologous recombination (HR) or nonhomologous end-joining (NHEJ) repair by targeting RAD51 or KU70/80 (Choi et al., 2014; Thapar, 2018). The presence of DNA lesions influences miRNA degradation as well as their expression. In both plants and animals, it has been demonstrated that miRNAs are responsive to irradiation (IR)-induced oxidative stress and may be responsible for the post-transcriptional regulation of some DDR genes (Joly-Tonetti and Lamartine, 2012; Kim et al., 2016). Plant specific miRNAs responsive to genotoxic stress include the IR-induced Arabidopsis miR840 and miR850, which remain to be further characterized in terms of their roles in DDR and DNA repair (Kim et al., 2016). Few rice miRNAs (osa-miR414, osa-miR164e, and osa-miR408) demonstrated to target specific helicases (Macovei and Tuteja, 2012) were also found to be responsive to γ-irradiation (Macovei and Tuteja, 2013). Predictive studies were employed as well; Liang et al. (2017) reported that MUTL-homolog 1 (MLH1) and MRE11 were putatively targeted by miR5176 and miR5261 in *Citrus sinensis* whereas the *Brachypodium distachyon* novel_mir_69 was identified to putatively target the RAD50 mRNA (Lv et al., 2016). Based on recent reviews of literature, an interrelation between DDR, redox systems, and miRNAs, has been proposed (Cimini et al., 2019). Nonetheless, specific hurdles have been pinpointed to explain the poorly represented examples in plants. Namely, this may be because DDR is significantly less studied in plants compared to animals (probably due to plant genome complexity) combined with limited information on miRNA targets specifically involved in coping with genotoxic stress (Chowdhury and Basak, 2019).

Considering the implications of DDR in plant genome stability, it is worth investigating deeper these fine-tuning aspects to gain novel insights on this complex topic. To address the existing gaps-of-knowledge, the current study proposes to explore the role of post-transcriptional regulation mediated by miRNAs in plant DDR. To do so, the first step consisted of setting up an original experimental system. This involved the administration of two compounds, namely, camptothecin (CPT, a well-known inhibitor of TopI enzyme) and NSC120686 (2-chloro-6-fluorobenzaldehyde 9H-fluoren-9-ylidenehydrazone). The latter was identified by Weidlich et al. (2010) as a substrate mimetic of the human TDP1. Together with topoisomerase inhibitors, NSC120686 has been used as a pharmacophoric model to suppress the TDP1 activity as part of a synergistic treatment for cancer therapies (Perego et al., 2012) whereas, in plants, dose-dependent genotoxicity was evidenced (Macovei et al., 2018a). As an experimental model, we have chosen to work on *M. truncatula*, because it is emerging as an informative and versatile system to investigate DDR during seed germination (Macovei et al., 2019). Moreover, DDR is an essential component of the seed repair response during germination (Waterworth et al., 2019) when active cell proliferation is determinant for the development of healthy seedlings and DNA damage must be repaired before the start of cell division to ensure the generation of robust plants. Phenotypic (germination percentage and speed, seedling growth) and molecular (cell death, DNA damage, and gene expression profiles) analyses demonstrated that the imposed treatments impact DDR. Subsequently, a list of miRNAs and putative target genes identified in a previous bioinformatics approach as being involved in DDR-associated biological processes (Bellato et al., 2019), were investigated in the developed system in terms of expression profiles. The results hereby presented show that miRNA/target gene expression is treatment-specific, thus confirming that miRNAs can be affected by DNA damage and that their targeted genes may have a contribution in the response to DNA damage.

## Materials and Methods

### Experimental Design

*Medicago truncatula* (cv. Jemalong) seeds, kindly provided by Fertiprado L.d.a. (Portugal), were used for this study. Seeds were treated with 25 μM CPT (Sigma-Aldrich, Milan, Italy), and 25 μM NSC120686 (NSC) provided by the National Cancer Institute (Bethesda, United States). A combined CPT + NSC treatment was implemented as well. The concentrations of the genotoxic agents were selected based on preliminary phenotypic results (Supplementary Figure S1) and previous studies (Macovei et al., 2018a). Because these compounds are dissolved in 100% dimethyl sulfoxide (DMSO, Sigma-Aldrich, Milan, Italy), specific DMSO controls, corresponding to each concentration used in the indicated treatments, were included. Specifically, DMSO 0.29% (subsequently denominated as DMSO_C) corresponds to the concentration used for the CPT treatments, DMSO 0.17% to NSC treatments (DMSO_N), and DMSO 0.23% to CPT + NSC treatments (DMSO_CN). The DMSO concentrations differ for CPT and NSC because the stock solutions (compounds dissolved in 100% DMSO) were prepared at different molarities (CPT 8.61 M and NSC 14.71 M), according to the manufacturer’s instructions. This affected also the combined treatment, where CPT and NSC were mixed 1:1. A non-treated control (NT) was used for all experiments. The designated treatments were applied to *M. truncatula* seeds placed in Petri dishes (30 seeds per dish) containing a filter of blotting paper moistened with 2.5 ml H_2_O (NT) or indicated solutions. Each sample/treatment was performed at least in triplicates. Petri dishes sealed with parafilm were kept in a growth chamber at 22°C under light conditions with a photon flux density of 150 μmol m^−2^s^−1^, photoperiod of 16/8 h, and 70–80% relative humidity. The experiment was followed for 7 days and subsequently, the harvested plant material was used fresh or frozen in liquid nitrogen (N_2_) for designated analyses.

### Phenotypic Evaluation

Treated and non-treated *M. truncatula* seeds were monitored for 7 days and data concerning germination percentage (%) and speed (T_50_), seedling length, and fresh weight (FW) were determined at the end of the experiment. The germination % parameter was assessed as the percentage of total germinated seeds in which the radicle protrusion reaches at least 1 mm of length. The time required for 50% of seeds to germinate (T_50_) was calculated according to the formula developed by Farooq et al. (2005): T_50_ = *t*_i_ + [(*N*/2 − *n*_i_) (*t*_i_ − *t*_j_)]/*n*_i_ − n_j_, where *N* is the final seed germination, *n_i_*, *n_j_* represent the cumulative number of seeds that germinated by adjacent counts at times *t_i_* and *t_j_* when *n*_i_ < *N*/2 < *n*_j_. Seedling length (millimeters, mm) was measured using millimetric paper whereas FW (grams, g) was measured using an analytical weight scale (Mettler AJ100, Mettler Toledo, Germany). Data are represented as mean ± SD of at least three independent measurements.

### Single Cell Gel Electrophoresis

The single cell gel electrophoresis (SCGE) protocol was implemented to *M. truncatula* radicles as previously described (Pagano et al., 2017; Araújo et al., 2019). Nuclei were extracted from treated/untreated radicles isolated from freshly harvested 7-day-old seedlings. For nuclei extraction, liquid N_2_ frozen radicles in Tris HCl EDTA (0.4 M Tris HCl pH 7.0, 1 mM EDTA pH 8) were finely sliced. The solution containing extracted nuclei was mixed with 1% low melting point (LMP) agarose and pipetted onto glass slides previously coated with 1% LMP. For alkaline SCGE, the glass slides containing isolated nuclei were subjected to electrophoresis (25 V, 300 A) in an alkaline buffer (0.3 M NaOH, 1 mM EDTA, and pH > 13) for 25 min at 4°C. For neutral SCGE, the slides were subjected to electrophoresis (20 V, 10 mA) in Tris-borate-EDTA (TBE; 89 mM Tris Base, 89 mM Boric Acid, 2 mM EDTA, and pH 8.3) for 8 min at 4°C. Subsequently, the slides were washed twice with Tris-HCl pH 7.5 for 5 min and rinsed in 70% ethanol (v/v) for 12 min. For nuclei count, the slides were stained with 20 μl 4',6- diamidino-2-phenylindole (DAPI, 1 μgml^−1^ stock solution; Sigma-Aldrich) and visualized at a fluorescence microscope (Olympus BX51, Olympus, Germany) with an excitation filter of 340–380 nm and a barrier filter of 400 nm. For each slide, about 100 nuclei were scored and analyses were performed in triplicates. The results were expressed in arbitrary units (a.u) calculated according to the formula proposed by Collins (2004): [*Σ*(*N*_c_ × *c*) × 100]/*N*_tot_, where *N_c_* is the number of nuclei of each class, *c* is the class number (e.g., 0, 1, 2, 3, and 4), and *N_tot_* is the total number of counted nuclei.

### DNA Diffusion Assay

The DNA diffusion assay was performed to evaluate cell death events and distinguish cells subjected to PCD or necrosis from viable cells as described by Macovei et al. (2018b). Nuclei extraction was performed from radicles of 7-day-old seedlings using the same methodology described for SCGE. The glass slides containing nuclei embedded in 1% LMP agarose were incubated in high salt lysis buffer (2.5 M NaCl, 100 mM EDTA, 10 mM Tris-HCl, and pH 7.5) for 20 min at 4°C to disrupt the nuclear membrane and permit DNA diffusion. The slides were immersed in neutral TBE for 5 min for three consecutive times to remove lysis solution and rinsed in 70% ethanol for 5 min at 4°C. Following DAPI staining, about 100 nuclei were scored (in triplicate samples) under the fluorescent microscope. The overall cell death level is given as a.u. while an additional analysis was used to represent the percentage of each class of nuclei (0-nuclei from viable cells, 1-nuclei from PCD cells, and 2-nuclei from necrotic cells).

### RNA Extraction, cDNA Synthesis, and Quantitative Real-Time PCR

Total RNA was isolated from treated and untreated *M. truncatula* seedlings as described (Pagano et al., 2017; Araújo et al., 2019). Briefly, liquid N_2_ grinded material was mixed with 550 μl Extraction Buffer (0.4 M LiCl, 0.2 M Tris pH 8.0, 25 mM EDTA, and 1% SDS) and 550 μl chloroform. Samples were centrifuged at 10,000 rpm for 3 min at 4°C. A phenol-chloroform solution was added to the supernatant followed by same centrifuge step. A 1/3 volume of 8 M LiCl was added to the supernatant, incubated at 4°C for 1 h, and subsequently centrifuged. The resulting pellet was washed with 70% ethanol, air-dried, and suspended in diethylpyrocarbonate (DEPC) water. The RNA was subsequently treated with DNase (Thermo Scientific), as indicated by the manufacturer. Finally, RNA was quantified with a NanoDrop spectrophotometer (Biowave DNA, WPA, ThermoFisher Scientific).

The complementary DNAs (cDNAs) were obtained using the RevertAid First Strand cDNA Synthesis Kit (ThermoFisher Scientific) according to the manufacturer’s suggestions.

The quantitative real-time PCR (RT-*q*PCR) reactions were performed with the Maxima SYBR Green qPCR Master Mix (2X; ThermoFisher Scientific) according to the supplier’s indications, using a Rotor-Gene 6000 PCR apparatus (Corbett Robotics Pty Ltd., Brisbane, Queensland Australia). Amplification conditions were as follows: denaturation at 95°C for 10 min, and 45 cycles of 95°C for 15 s and 60°C for 60 s. Oligonucleotide primers (Supplementary Table S1) were designed using Primer3Plus^1^ and verified with Oligo Analyzer.^2^ The relative quantification was carried out using actin-related protein 4A (*Act*) and elongation factor 1α (*ELF1α*) as reference genes since they resulted the most stable under the tested conditions following geNorm (Vandesompele et al., 2002) analysis (Supplementary Figure S2). The raw, background-subtracted fluorescence data provided by the Rotor-Gene 6000 Series Software 1.7 (Corbett Robotics) was used to estimate PCR efficiency (E) and threshold cycle number (C_t_) for each transcript quantification. The Pfaffl method (Pfaffl, 2001) was used for the relative quantification of transcript accumulation. All reactions were performed in triplicate. The data are presented as fold change (FC), where values for each treatment were normalized to their corresponding DMSO control. Heatmaps were constructed using the Shinyheatmap tool (Khomtchouk et al., 2017).

### microRNAs Expression Analysis

For miRNAs expression, total RNA was isolated using TRIzol (ThermoFisher Scientific), as indicated by the supplier. The two-tailed RT-*q*PCR technique (Androvic et al., 2017) was performed to quantify miRNA accumulation. The miRNAs expression profiles were analyzed in 7-day-old untreated and treated seedlings. Different sets of primers were used to perform reverse transcription (RT) and RT-*q*PCR for each mature miRNA, one to synthesize the cDNA and two for the SYBR *q*PCR amplification. cDNAs were obtained using the qScript® Flex cDNA Synthesis Kit (QIAGEN, Beverly, Massachusetts). The RT primers (Supplementary Table S2) were designed to have a two-tailed structure as indicated by Androvic et al. (2017). RNAfold WebServer^3^ was used to predict the stable secondary structure. To obtain the cDNA, a forward primer specific for the designed region in the 5'-terminus of the two-tailed RT-primer and a reverse primer specific for the miRNA target sequence were used. Subsequently, RT-*q*PCR was performed as described in the above paragraph using the oligonucleotide primers shown in Supplementary Table S3.

### Statistical and Integrative Data Analyses

For phenotypic evaluation, the significance of mean differences was determined using the Student’s *t*-test. For gene/miRNA expression data, following the normality test (Shapiro-Wilk), a one way ANOVA on ranks was performed using the Kruskal-Wallis test in an R (software version 4.0.2) background.

Principal components analysis (PCA) was performed on the phenotypic and molecular variables quantified across the study using the FactoMineR (Lê et al., 2008) and factoextra (Kassambara and Mundt, 2020) packages in R environment for statistical computing and graphical design. Values were standardized by means of *z*-score using the default scaling settings of the PCA function. The included variables were: germination %, T_50_, seedling length (divided as aerial part and radicles), DNA damage levels, all gene expression data, and miRNA expression profiles.

## Results

### CPT and NSC Treatments Do not Affect Seed Germination but Impair Seedling Development

The CPT and NSC120686 inhibitors require to be dissolved in DMSO, which, at certain concentrations, can impair plant development (Zhang et al., 2016). Thus, it was necessary to first identify the inhibitor concentrations at which minimal or null DMSO effects are evident at a phenotypic level. In the case of the CPT treatments, the selected concentration was 25 μM dissolved in 0.29% DMSO (Supplementary Figure S1). The selection of NSC concentration (25 μM dissolved in 0.17% DMSO) was based on previous results (Macovei et al., 2018a). The last treatment consisted of synergistically exposing *M. truncatula* seeds to CPT 25 μM and NSC 25 μM (treatment denominated as CPT + NSC), dissolved in 0.23% DMSO. As described in “Materials and Methods,” each corresponding DMSO concentrations (denominated as DMSO_C, DMSO_N, and DMSO_CN) were tested along with the non-treated (water) control (NT).

**Figure 1:**
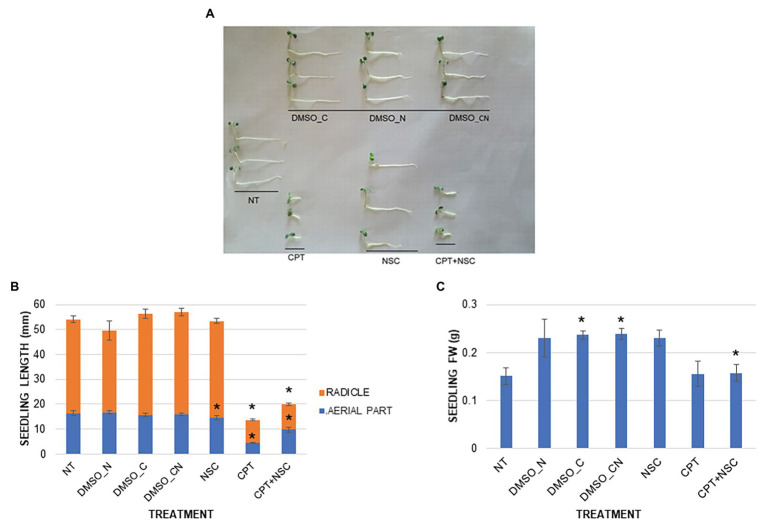
Phenotypic effect of CPT, NSC, and CPT + NSC treatments, and corresponding DMSO concentrations (DMSO_N, DMSO_C, and DMSO_CN) on Medicago truncatula seed germination. **(A)** Representative image of 7-day-old seedlings. **(B)** Seedling length (mm). **(C)** Fresh weight, FW (g). Data are represented as mean ± SD of three independent replicates. Statistically significant (*p* < 0.05) differences between treatments and control (NT) are represented with an asterisk (^*^). CPT, camptothecin; NSC, TDP1 inhibitor NSC120686; and DMSO, dimethyl sulfoxide.

To verify whether CPT and NSC influence seed germination, a phenotypic characterization was performed by evaluating germination % and speed (T_50_), seedling length, and FW after 7 days of treatment. While seed germination % and T_50_ were not significantly affected by any of the imposed treatments at the end of the indicated timeframe (Supplementary Figure S3), CPT impacted mostly on seedling development. Figure 1A shows the morphology of the 7-day-old seedlings, grown in the presence of CPT, NSC, and CPT + NSC, and their corresponding DMSO controls. Treatment with the NSC inhibitor did not result in a visible change in seedling morphology while seedlings treated with CPT and CPT + NSC appeared shorter and stockier than the relative controls. These observations are supported by the registered significant (*p* < 0.05) differences when measuring the seedling length and FW (Figure 1B). A reduction in seedling length was caused by the CPT and CPT + NSC treatments, with radicles being more severely affected than the aerial parts. A minor, although still significant impact, was observed in the case of the NSC-treated seedlings. When considering the FW parameter, an increase in seedling weight was detected for DMSO_C and DMSO_N, while FW was significantly decreased in the NSC + CPT-treated seedlings (Figure 1C).

Overall, these results show that the imposed treatments do not affect germination *per se* but inhibit seedling growth, especially in the CPT- and CPT + NSC-treated samples. This may lead to assume that CPT contributes the most to the impairment of the seedling growth since a lesser effect was observed when the NSC compound was delivered alone.

### The Imposed Treatments Induce Different Cell Death Profiles

**Figure 2:**
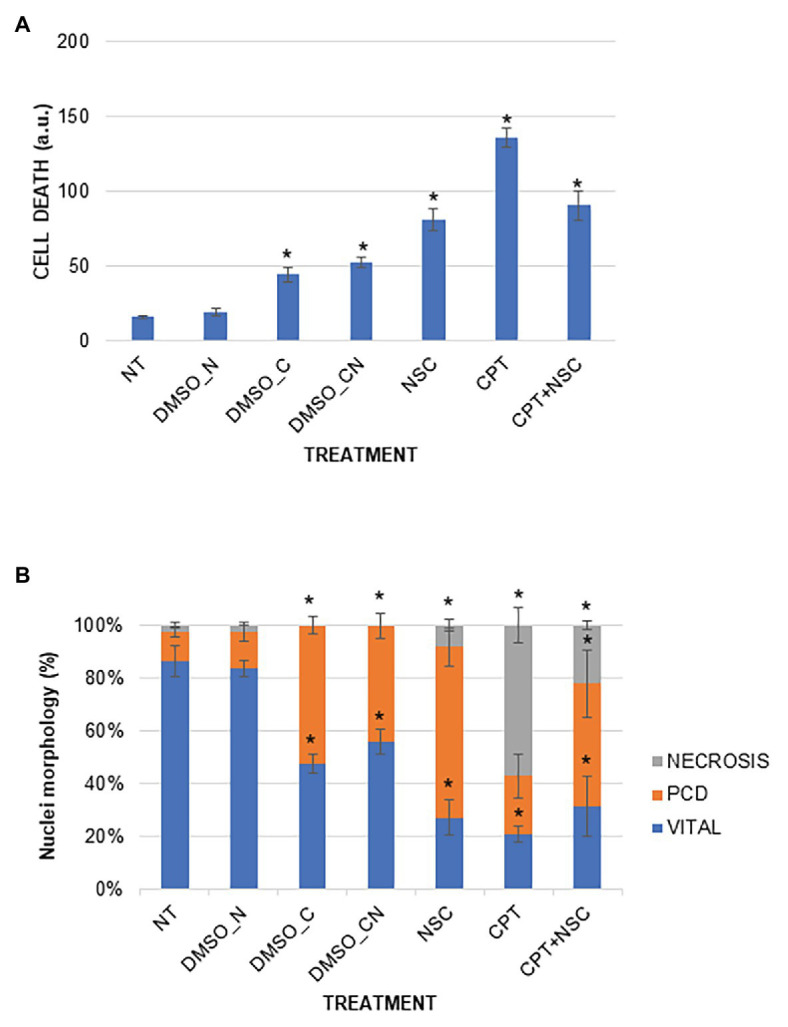
Cell death induced by the imposed treatments in *M. truncatula* 7-day-old seedlings. **(A)** Overall cell mortality scores represented as arbitrary units (a.u.). **(B)** Cell death represented as percentage of nuclei per class. Values are expressed as mean ± SD of three replicates. Statistically significant (*p* < 0.05) differences between treatments and control (NT) are represented with an asterisk (^*^). CPT, camptothecin; NSC, TDP1 inhibitor NSC120686; and DMSO, dimethyl sulfoxide.

A DNA diffusion assay was performed to evaluate cell death events in 7-day-old *M. truncatula* seedlings subjected to CPT and NSC treatments (Figure 2). The results of the diffusion assay were expressed both as arbitrary units (a.u.) to indicate the overall level of cell death and as percentage of nuclei per class to indicate the different types of cell death events (class 0 – viable cells, class 1 – programmed cell death events, and class 2 – necrosis events). Enhanced levels of cell death are evident in the imposed treatments when compared to NT, with the highest values registered during the CPT treatment (Figure 2A). Cell death significantly increased also in samples treated with DMSO_C and DMSO_CN but at a substantially lesser degree than when compared to the CPT/NSC system. When looking at the different types of nuclei classes, the data show that the NT and DMSO_N samples are both characterized by a high percentage of viable nuclei (86.36 ± 6.00 and 83.63 ± 3.16%, respectively) and a low percentage of PCD and necrosis (Figure 2B). Seedlings treated with DMSO_C and DMSO_CN started to show a decrease in viable nuclei (47.60 ± 3.40, 55.74±4.74%) toward PCD, while the nuclei classified as belonging to necrotic cells (class 2) are not present. Class 2 nuclei are mostly present in CPT and CPT + NSC samples, while the NSC treatments evidence the presence of class 1 nuclei characteristic for PCD events (Figure 2B). Concerning the NSC- and CPT + NSC-treated samples, a marked decrease in the percentage of viable nuclei (27.18 ± 6.76, 31.52 ± 11.18%) is observed with a concomitant increase in the percentage of nuclei subjected to PCD (52.12 ± 5.49, 46.53 ± 12.7%) and necrosis (27.38 ± 6.20, 21.9 ± 6.20%). Similarly, a reduction in the percentage of viable nuclei is observed for CPT-treated samples (21.05 ± 2.91%), where the most represented nuclei belong to class 2 (57.13 ± 6.82%), characteristic for the presence of necrotic events.

Overall, the imposed treatments decrease cell vitality and induce different types of cell death events. The most severe effects are observed with the CPT treatment, characterized by a high level of necrosis whereas PCD events prevail in the NSC treatment. In the CPT + NSC combination, both PCD and necrosis events are registered at similar levels.

### The Imposed Treatments Cause Accumulation of DNA Damage

**Figure 3:**
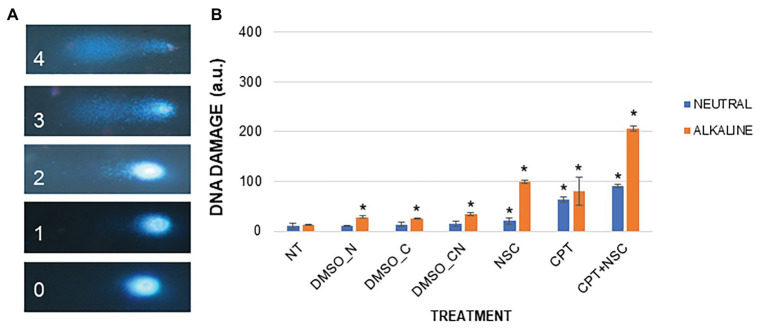
DNA damage induced by the imposed treatments in *M. truncatula* 7-day-old seedlings. **(A)** Nucleus morphology and its related class identification number. **(B)** DNA damage scores represented as a.u. Values are expressed as mean ± SD of three replicates. Statistically significant (*p* < 0.05) differences between treatments and control (NT) are represented with an asterisk (^*^). CPT, camptothecin; NSC, TDP1 inhibitor NSC120686; and DMSO, dimethyl sulfoxide.

To quantitatively measure DNA damage, SCGE was performed using both the alkaline and neutral versions of the assay. Representative images for each nuclei class (0–4) are provided (Figure 3A). The neutral version generally detects double-stranded breaks (DSBs) whereas the alkaline version includes different types of breaks such as single-strand breaks (SSBs) formed from alkali-labile sites, DNA-DNA, or DNA-protein cross-links (Ventura et al., 2013). Compared to NT, the NSC-treated samples showed a 7.22-fold increase in the level of DNA damage under alkaline conditions while only a 1.99-fold increase was observed under neutral conditions (Figure 3B). A 5.86- and 5.79-fold increase in the level of DNA damage was observed in the CPT-treated samples under alkaline and neutral conditions, respectively. The CPT + NSC-treated samples showed a 13.7-fold increase in the level of DNA damage in alkaline conditions while an 8.4-fold increase was detected under neutral conditions. Considering the DMSO controls, no significant differences in the accumulation of DNA damage as DSBs are evident under neutral conditions. However, a small but significant increase in the levels of DNA damage was registered under alkaline conditions. This may suggest that DMSO could generate SSBs, alkali-labile sites, incomplete excision repair sites, and DNA-DNA/DPCs rather than more extensive damage like DSBs.

Overall, the observed results indicate that the administration of CPT/NSC agents cause an accumulation of both SSBs and DSBs, but at different degrees depending on the type of treatment. While in the case of NSC, SSBs and associated damage types are prevalent, for the CPT treatments an additional increase in the presence of DSBs is observed. The combination of the two agents (CPT + NSC) resulted in the highest level of DNA damage, combining DSBs, SSBs, and associated damage, the latter being most prevalent.

### CPT/NSC Treatments Trigger Differential Expression of DDR-Related Genes

Given that CPT/NSC treatments resulted in reduced seedling growth, increased cell mortality, and accumulation of DNA damage, the next step consisted in the evaluation of DDR-related gene expression profiles using RT-*q*PCR. The following genes were selected:

1. *SOG1*, as the master-regulator of plant DDR;
2. *TDP1α*, *TDP1β*, *TDP2α*, *Top1α*, and *Top2*, as genes that encode for proteins most probably affected by the CPT and NSC inhibitors;
3. *MRE11*, *RAD50*, *NBS1*, *PARP1*, *ERCC1*, and *MUS81*, as genes that encode for proteins involved in repair processes considered as alternative to the function of *TDP1* genes. The genes belonging to the MNR complex were selected as they represent the frontline players in the detection and signaling of DSBs, thus HR and NHEJ repair pathways. On the other side, *PARP1*, *ERCC1*, and *MUS81* are associated with both BER and DPC repair. All selected genes were already validated in *M. truncatula* calli exposed to NSC120686 (Macovei et al., 2018a).
4. *TOR*, *CDKA1*, *CycB1*, *CycD2*, and *CycD3*, as genes that encode for proteins known to be involved in the regulation of the cell cycle.

**Figure 4:**
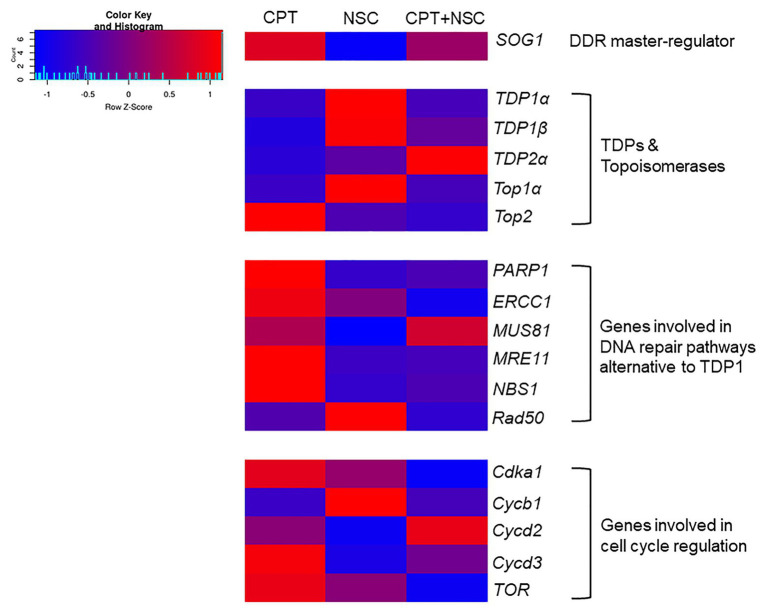
Heatmap representing fold changes (FCs) in gene expression values in response to CPT, NSC, and CPT + NSC treatments in 7-day-old M. truncatula seedlings. For each treatment, the values were normalized to their corresponding DMSO controls. The heatmap was constructed using the Shinyheatmap. CPT, camptothecin; NSC, TDP1 inhibitor NSC120686 (http://shinyheatmap.com/) application.

Because the expression of the genes appears to be influenced by DMSO (Supplementary Figure S4), and to evaluate the real effect that CPT and NSC treatments may induce at the level of gene expression, the data are presented as FC to control, where the control is represented by each corresponding DMSO concentration. The FC values were used to generate a heatmap (Figure 4), where blue color indicates downregulated genes and red color indicates upregulated genes compared to their respective controls. The ANOVA analysis show statistical differences (*p* < 0.05) between treatments and controls for the majority of investigated genes (Supplementary Table S4). These results show that the *SOG1* gene is upregulated by CPT and downregulated by NSC, suggesting a contrasting effect of the two treatments at the level of DDR. This contrasting trend is maintained as well when looking at the expression levels of most investigated genes. *TDP1α*, *TDP1β*, and *TopIα* are upregulated by NSC and downregulated by CPT treatments. Conversely, most of the genes involved in alternative DNA repair pathways (*PARP1*, *ERCC1*, *MUS81*, *MRE11*, and *NBS1*) are upregulated by CPT and downregulated by NSC treatments. Within the genes involved in the regulation of the cell cycle, *Cdka1*, *Cycd3*, and *TOR* are upregulated during CPT treatments whereas *Cycb1* is upregulated by NSC. The concomitant administration of CPT + NSC had a different response compared to the individual CPT or NSC treatments; namely, most of the investigated genes are downregulated and the only upregulated genes are *TDP2α*, *MUS81*, and *Cycd2*.

Overall, the gene expression data indicate a contrasting effect for the single administration of NSC and CPT treatments along with a distinct response in case of the synergistic exposure to both compounds where most investigated genes appeared downregulated.

### Expression Analyses of Selected microRNAs and Their Putative Targets

Since the main goal of this work was to identify miRNAs able to regulate DDR-associated processes, we proceeded with the investigation of different miRNA-target gene pairs, previously identified from bioinformatics analyses as being related to DDR processes (Bellato et al., 2019). The expression profiles of selected miRNAs and putative target genes were investigated in the CPT/NSC system, proven to affect DDR. Specifically, the following miRNA-gene pairs were considered:

1. mtr-miR156a, identified as putatively targeting *UBE2A* (ubiquitin-conjugating enzyme, Medtr4g108080), involved in histone modification processes.
2. mtr-mir172c-5p, putatively targeting *RAD54-like* (DNA repair and recombination RAD54-like protein, Medtr5g004720), involved in DSBs repair.
3. mtr-miR2600e, putatively targeting *5AT* (anthocyanin 5-aromatic acyltransferase, Medtr2g089765), involved in antioxidant defense.
4. mtr-mir395e, putatively targeting *DMAP1* (DNA methyltransferase 1-associated protein, Medtr1g086590), associated with histone modifications.
5. mtr-miR5741a, putatively targeting *E2FE-like* (E2F transcription factor-E2FE-like protein, Medtr4g106540), involved in DNA-dependent DNA replication.
6. mtr-miR168a, targeting *AGO1A* (Argonaute protein 1, Medtr6g477980), used as a control since the relation between this miRNA and target gene has already been experimentally validated (Vaucheret et al., 2004, 2006).

**Figure 5:**
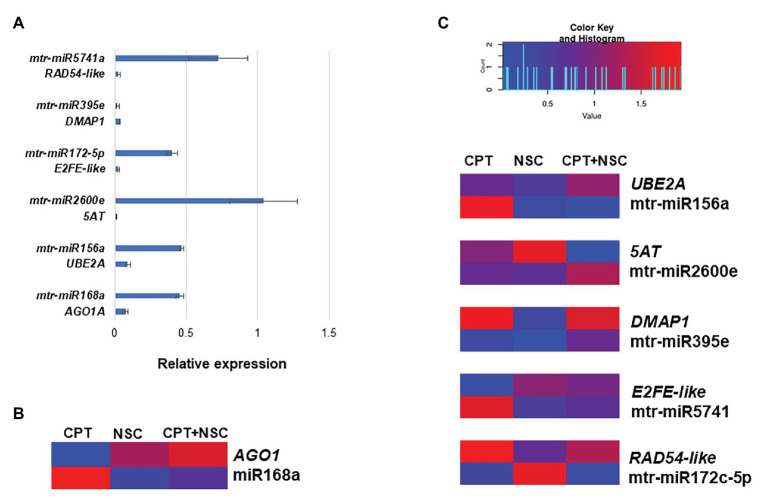
Expression profiles of selected miRNAs and their putative targeted genes in 7-day-old *M. truncatula* seedlings. **(A)** Relative expression of miRNAs/genes pairs in non-treated (NT) samples. **(B)** *AGO1A* and mtr-miR168a pair used as data quality control. **(C)** Heatmap representing fold changes (FCs) to each corresponding DMSO of miRNAs and putative targeted genes in response to CPT, NSC, and CPT + NSC treatments. The heatmap was constructed using the Shinyheatmap (http://www.shinyheatmap.com/) application. CPT, camptothecin; NSC, TDP1 inhibitor NSC120686

The expression profiles of miRNAs and putative target genes are shown in Figure 5 while associated statistics are given in Supplementary Table S4. First, their expression in non-treated (NT) samples was monitored to evaluate their behavior under physiological conditions. As shown in Figure 5A, while the majority of the tested miRNAs are highly expressed (except for mtr-miR395e), the expression of their putative target gene is significantly reduced, thus corroborating the expected trend where miRNAs activity inhibits the expression of the targeted gene. The ability of miR168a to target *AGO1A* gene is a well-known fact to the scientific community (Vaucheret et al., 2004, 2006), therefore, this miRNA was chosen as quality control for function/target validation. Indeed, a low level of *AGO1A* expression corresponds to a high level of miR168a expression in NT samples (Figure 5A). Looking into the expression of this specific miRNA and its target gene during the imposed treatments, it is evidenced that when the expression of miR168 is low, the expression of *AGO1A* is high, and vice-versa (Figure 5B).

Since gene expression is influenced by DMSO, also in this case, data are represented as FC to respective controls and gathered in a heatmap (Figure 5C) where blue color represents downregulation and red color represents upregulation. Overall, the heatmap shows that under most conditions when a miRNA is upregulated the corresponding candidate target gene is downregulated. Looking at the miRNAs expression according to each treatment, it is possible to observe a treatment-specific behavior where different miRNAs expression is triggered by different treatments. Namely, mtr-miR156a and mtr-miRA5671 are upregulated by CPT, mtr-miR172c-5p is upregulated by NSC, and mtr-miR2600e are upregulated by CPT+NSC.

Overall, an indirect evidence of miRNA action over these targets is provided; the contrasting profiles between miRNA-predicted target abundances support the evidence that these miRNAs could repress the expression of these targets.

### Principal Component Analysis for Data Integration

**Figure 6:**
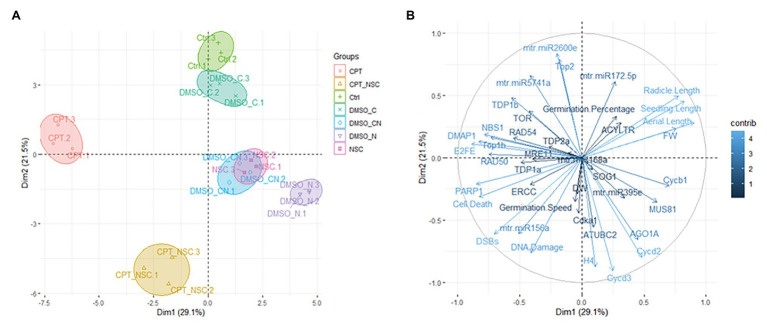
Principal component analyses (PCA). **(A)** Loading plot explaining the distribution of samples focusing on the imposed treatments. **(B)** Loading plot explaining the contribution of each measured variable (germination %, T_50_, seedling length, FW, gene, and miRNAs expression profiles).

Principal components analysis was used to investigate the differences between samples and which variables most contributed to these differences (Figure 6). The *X*-axis and *Y*-axis show the principal dimension Dim1 and Dim2 that explain 29.1 and 21.5% of the total variance, respectively. Prediction ellipses are such that with probability 0.95, a new observation from the same group will fall inside the ellipse. The orientation of the ellipses shows that the most different samples are those treated with CPT and CPT + NSC whereas the NSC treatment is located in the proximity of DMSO_CN- and DMSO_N-treated samples (Figure 6A). Other distinctive groups are formed by the NT and DMSO_C samples located in the upper-right panel. Hence, the plotted data allow a clear separation of the majority of the samples according to the imposed treatments. The observed vicinity among replicates is indicative of data reliability. The variables that most contributed to the group differentiation are represented in a light blue color (Figure 6B). Among the phenotypic parameters, the most representative variables include seedling length, cell death, and DNA damage. Amidst the investigated genes, DMAP1, E2FE-like, PARP1, Cycd3, and Cycd2 had the highest contribution but also TDP1β, Top1α, Top2, and NBS1 are well-represented. When considering the miRNAs, it is relevant to underline that these had an important contribution to the differentiation of the samples and the most representative ones are mtr-miR2600e and mtr-miR5741.

## Discussion

In this work, CPT and NSC120686 were used alone or in combination to develop an original experimental system in which plant specific DDR functions would be altered so that miRNAs associated with DDR pathways could be revealed. CPT is a widely used agent much employed in anticancer therapies due to its activity as TopI inhibitor since it intercalates between DNA breaks flanking the TopI-cleavage complex (Pommier et al., 2010). CPT is known for its cytotoxic effects also in plants (Buta and Worley, 1976; Takahashi et al., 2002) where enhanced levels of cell death had been registered (Locato et al., 2006; Iakimova et al., 2020) presumably through the accumulation of TopI-covalent complexes as in the case other eukaryotes. On the other side, the NSC120686 compound was recently identified based on virtual screening of pharmacophores able to inhibit human Tdp1 (Weidlich et al., 2010) and subsequently used in combination with CPT-derivates to inhibit the growth of different cancer cell lines (Perego et al., 2012). *Medicago truncatula* calli treated with different concentrations of NSC120686 displayed enhanced levels of cell mortality and DNA damage (Macovei et al., 2018a). So far, combined application of the two agents has not been reported in plants.

Before evaluating the possible involvement of miRNAs in this system, it was first necessary to prove that it targets DDR-associated processes. The phenotypic investigation revealed that CPT and CPT + NSC had a major effect on seedling development mostly by inhibiting radicle growth while the single administration of NSC had a milder effect (Figure 1). Hence, the phenotypic changes could be mostly attributed to CPT, as in agreement with previous studies, where 25 μM of CPT substantially inhibited the growth of *Arabidopsis* plantlets while concentrations higher than 50 μM resulted in a strong impairment of both roots and shoots at young seedling stages (Takahashi et al., 2002). In accordance with the observed phenotypes, enhanced levels of cell death and accumulation of DNA damage were evidenced (Figures 2, 3). Interestingly, different types of cell death events and DNA damage were encountered according to the imposed treatments. While CPT administration resulted in enhanced levels of necrosis and accumulation of DSBs, the delivery of NSC was accompanied by PCD and accumulation of SSBs, DNA-DNA, or DPCs. For the CPT + NSC combination, both PCD and necrosis events are present at similar levels while the high levels of DNA damage indicate the most genotoxic effect. Previous literature reports that CPT results in the accumulation of DPCs (Enderle et al., 2019b) and DSBs (Ferrara and Kmiec, 2004; Berniak et al., 2013), lesions know to be associated with necrotic events in plant cells (Rowan et al., 2010; Song and Bent, 2014). On the other hand, low concentrations of NSC120686 resulted in enhanced levels of PCD in *M. truncatula* calli (Macovei et al., 2018a).

**Figure 7:**
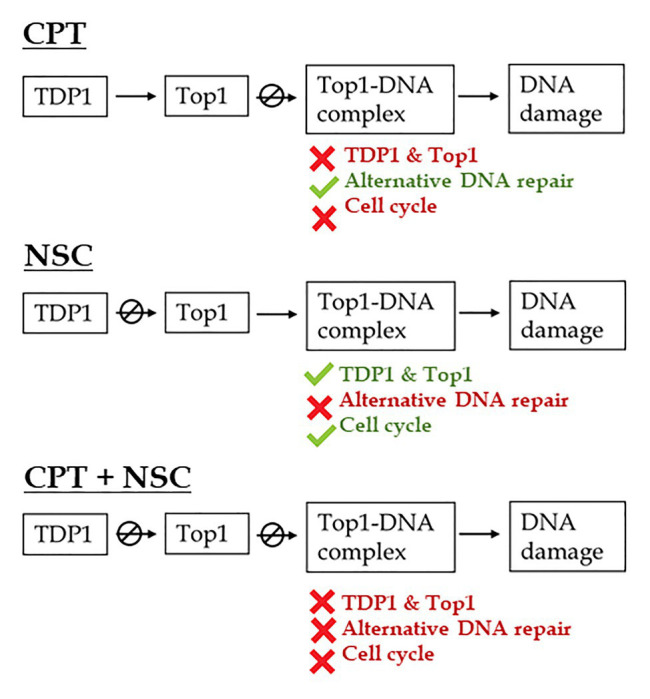
Schematic representation of the proposed effects of CPT and NSC, inhibitors of Top1 and TDP1 enzymes, on DNA repair pathways and cell cycle regulation during M. truncatula early seedling development. CPT, camptothecin; NSC, TDP1 inhibitor NSC120686.

The outlined distinction between treatments was maintained when considering the expression profiles of selective genes belonging to different DNA repair pathways and cell cycle regulation (Figure 4). In addition to *TDP1*, *α*, and *β*, and *Top1α* genes, *TDP2α*, and *Top2* genes were investigated because of the closed connection between these two, as TDP2 enzyme is involved in the removal of DNA TopII-mediated DNA damage and cell proliferation/differentiation signaling (Cortes Ledesma et al., 2009). Moreover, the overexpression of *TDP2α* gene in *M. truncatula* was correlated with a decrease in the accumulation of DSBs, increased cell proliferation, and enhanced resistance to stress (Confalonieri et al., 2014; Faè et al., 2014; Araújo et al., 2016). Genes involved in DNA repair alternative to TDP1 (Pommier et al., 2014) include the MNR complex, composed of *MRE11*, *NBS1*, and *Rad50*, known to be involved in the detection of DBSs and HR (Manova and Gruszka, 2015) as well as *PARP1*, *MUS81*, and *ERCC1* involved in BER and DPC repair (Enderle et al., 2019b; Roldán-Arjona et al., 2019). Since DDR includes a response from both DNA repair and cell cycle regulation, several cyclins (*Cdka1*, *Cycb1*, *Cycd2*, and *Cycd3*) were investigated alongside the master-regulators *TOR* and *SOG1*. The observed changes in the expression profiles of *SOG1* gene indicate that DDR is truly affected by the imposed treatments; hence, we can conclude that the developed system has an impact on DDR. To briefly summarize the behavior of the tested genes in association with the phenotypic observations, the following assumptions are taken into consideration (Figure 7):

1. During the CPT treatment, TopI enzyme is presumably blocked, TopI-DNA covalent complexes would accumulate and high levels of DNA damage and cellular mortality are registered, resulting in substantial inhibition of seedling growth. In this situation, *TDP1* and *Top1* genes are inhibited while genes involved in DNA repair pathways alternative to TDP1 are highly active. Based on the expression of genes involved in the cell cycle, this is delayed presumably to allow the repair of the induced DNA damage.
2. When NSC is given, the TDP1 enzyme would interact with this mimicking compound, thus being prevented from engaging with its substrate and hydrolyze the crosslink between TopI and DNA. In turn, this may again lead to the accumulation of these complexes and the subsequently observed enhancement in the levels of cell death and DNA damage, although at a lesser extent, in agreement with the phenotypic observations. In this case, the *TDP1* and *Top1* genes are active, the alternative DNA repair is inhibited, and the cell cycle is progressing.
3. The CPT + NSC combination may target both TDP1 and TopI functions and this leads to the highest cytotoxic and genotoxic effects, corresponding to the obstructed seedling development. In terms of gene expression, this treatment induced the downregulation of most of the investigated genes, affecting both DNA repair and cell cycle progression.

In a previous bioinformatics investigation, we have identified specific miRNAs (mtr-miR156a, mtr-mir172c-5p, mtr-miR2600e, mtr-mir395e, and mtr-miR5741) putatively targeting genes associated with DDR processes (Bellato et al., 2019). Among these, miR156 is an evolutionarily conserved family, although diversification in its members, sequence, and functions are present (Sunkar and Jagadeeswaran, 2008; Cui et al., 2017). Others, like miRNA172 family has been associated with seed development alongside with other regulatory functions (Smoczynska and Szweykowska-Kulinska, 2016). High-throughput sequencing of *M. truncatula* seedlings found that miR156 and miR172 are involved in salinity stress (Cao et al., 2018). MiR395 is involved in sulfate assimilation regulatory network (Matthewman et al., 2012) whereas miR5741 has been associated with roles in the defense response (Siemens et al., 2006). It is therefore clear that these miRNAs have been studied mainly in relation to plant development and response to biotic/abiotic stress. The RT-*q*PCR analyses performed in this work indicate that they are also involved in the response to genotoxic stress, as indicated by their differential expression induced by the CPT/NSC treatments. For example, mtr-miR172c-5p is upregulated in NSC treated samples and downregulated in CPT treated samples. By observing the expression profiles of its putatively targeted gene *E2FE-like*, it is shown that an upregulation of the miRNA is accompanied by a downregulation of the gene predicted to be its target. Importantly, this gene is a homolog of the *Arabidopsis* E2F transcription regulator shown to be involved in cell cycle regulation, DNA replication, and DNA damage repair, in pathways alternative to SOG1 (Berckmans and De Veylder, 2009; Roa et al., 2009; Gutzat et al., 2012).

In conclusion, by inducing seedling growth inhibition, accumulation of cell death, and DNA damage, along with the differential expression of genes involved in DDR, the developed CPT/NSC system actively influence DDR-associated processes. Above all, we demonstrated that specific miRNA-target gene pairs, identified from a bioinformatics approach, are responsive to the imposed treatments, thus showing that these miRNAs have a role to play in DDR. This study extends the knowledge regarding the roles played by miRNAs in the post-transcriptional regulation of DDR in plants. This may disclose new regulatory networks with further possibilities regarding biotech application relevant to enhance crop adaptation to genotoxic stresses. Given the complexity of regulatory networks and since miRNAs can repress multiple targets, further functional validation studies are needed to corroborate these suggested roles in DDR. This is particularly relevant to clarify if other regulatory mechanisms might be responsible for the observed downregulation of target genes expression.

## Data Availability Statement

The raw data supporting the conclusions of this article will be made available by the authors, without undue reservation.

## Author Contributions

AM conceptualized the study. CG, MG, AP, and TC performed the treatments and conducted the designed analyses. AM, MG, CG, and SA analyzed and interpreted the data. AM and AB wrote the manuscript. All authors contributed to the article and approved the submitted version.

### Conflict of Interest

The authors declare that the research was conducted in the absence of any commercial or financial relationships that could be construed as a potential conflict of interest.

## Footnotes

^1^https://primer3plus.com/

^2^https://eu.idtdna.com/calc/analyzer

^3^http://rna.tbi.univie.ac.at//cgi-bin/RNAWebSuite/RNAfold.cgi
