# Supplementary material for: Exploring microRNA Signatures of DNA Damage Response Using an Innovative System of Genotoxic Stress in Medicago truncatula Seedlings
Source: Front Plant Sci. 2021 Mar 9;12:645323. doi: 10.3389/fpls.2021.645323 (PMC7985446; doi:10.3389/fpls.2021.645323)
Supplement: Supplementary file 1 [file Data_Sheet_1.docx]

Supplementary Material

- 1. **Selection of CPT concentration**

Three different concentrations of CPT (25 μM, 50 μM, and 100 μM), along with their corresponding DMSO concentrations (0.29%, 0.58%, and 1.16%,) were tested during *M. truncatula* seed germination. Seeds were considered germinated when radicle protrusion reaches at least 1 mm. Biometrical analyses, consisting of measuring the seed germination percentage (%) and speed (T_50_), seedlings length, and fresh weight (FW), were used to establish the phenotypic effect of CPT and DMSO after 7 days of treatment (**Supplementary Figure 1**). No significant differences (*P* > 0.05) between NT and treatments were observed regarding germination percentage (**Supplementary Figure 1A**), as germination rates varied between 44.4±31.68% (DMSO 1.16%) and 82.2±8.39% (NT). Similarly, regarding germination speed, most seeds germinated within the first two days (**Supplementary Figure 1B**). These results indicate that the tested CPT and DMSO concentrations do not affect germination percentage or speed. The high standard deviation values indicate that the germination is not uniform. However, growth inhibition was observed in seedlings at the end of the 7th day. A significant (*P* < 0.05) decrease in seedling length is evident when comparing NT (56.27±1.35 mm) with CTP 25 μM (13.73±0.19 mm), CTP 50 μM (12.87±0.46 mm), and CTP 100 μM (12±0.17 mm) treatments (**Supplementary Figure 1C**). Among the tested DMSO concentrations, no significant negative effect was encountered. When considering the seedling FW, no significant differences were observed between NT and treatments (DMSO, CTP), as all samples weighted between 0.12 - 0.22 g (**Supplementary Figure 1D**).

These analyses allowed the identification of a CPT concentration able to cause an evident phenotypic effect considering also its corresponding DMSO concentration in such a way to not cause any phenotypic effect on seedling growth. Hence, the concentration of CPT that met these requirements was 25 μM dissolved in 0.29% of DMSO. The subsequent experiments were carried out using this concentration.


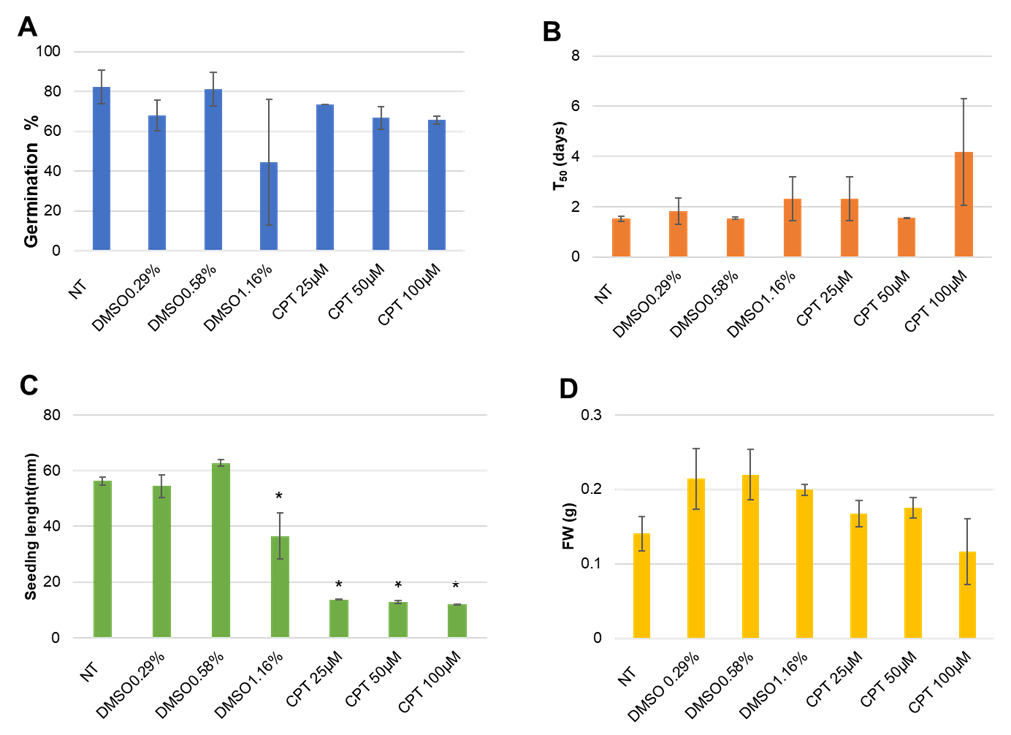


**Supplementary Figure 1.** Phenotypic effect of camptothecin (CPT) and dimethyl sulfoxide (DMSO) on *Medicago truncatula* seed germination and seedling establishment. (**A**) Germination percentage (%). (**B**) Germination speed, T_50_ (days). (**C**) Seedling length (mm). (**D**) Fresh weight, FW (g). Data is collected after 7-days of treatment and represented as mean ± standard deviation of at least three independent replicates. Statistically significant (*P*<0.05) differences between treatments and non-treated control (NT) are represented with an asterisk (*).

- 1. **Selection of most stable reference genes**

Before starting the experiment, it was necessary to identify the most stable reference genes under the imposed conditions to be used for the relative quantification of the transcripts. To this purpose, a geNorm analysis was carried out to evaluate the stability of the following endogenous genes: *PDF2* (protodermal factor 2), *GAPDH* (glyceraldehyde-3-phosphate dehydrogenase), *PPRep* (pentatricopeptide repeat), *TUB* (tubulin), *Ubi* (ubiquitin), *Act* (actin), and *Elf1α* (elongation factor 1α). The expression level of these six internal control genes was evaluated in the seven samples derived from non-treated seedlings, DMSO-treated, and CPT/NSC-treated samples. GeNorm is a popular algorithm used to determine the most stable reference genes from a set of tested candidate reference. The program enables the elimination of the worst-scoring housekeeping gene (the one with the highest M value) and recalculation of new M values for the remaining genes to determine the most stable gene (the one with the lower M value). The results of this analysis showed that *Act* and *Elf1α* had the most stable expression under the imposed conditions (**Supplementary Figure 2**).


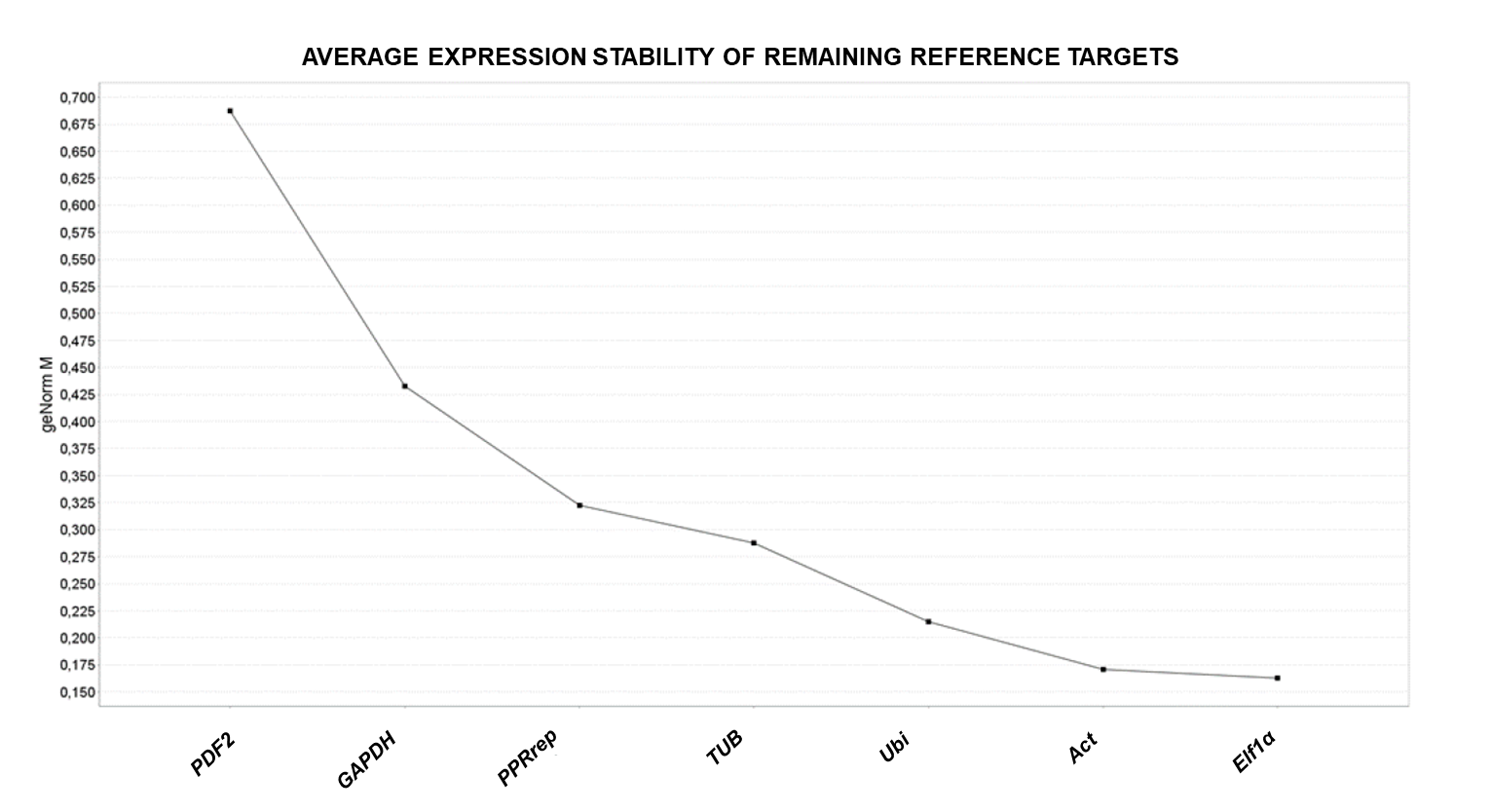


**Supplementary Figure 2**. Selection of reference genes for qRT-PCR analysis. Average expression stability values (geNorm M), calculated using GeNorm algorithm (https://genorm.cmgg.be), of the seven reference genes analysed in *Medicago truncatula* treated/non-treated seedlings as indicated in the experimental design. PDF2, protodermal factor 2; GAPDH, glyceraldehyde 3-phosphate dehydrogenase; PRRrep, pentatricopeptide (PPR) repeat; TUB, tubulin; Ubi, ubiquitin; Act, actin; Elf1α, elongation factor 1α.

- 1. **The effect of CPT/NSC treatments on seed germination**

When comparing the germination percentage of the non-treated (NT, 60.00±13.33%) with treated samples, a significant (*P* < 0.05) decrease was observed only on the first day for the DMSO_C (16.66±12.01%) and CPT+NSC (4.44±5.09%) treatments. No significant differences were observed during the following days until the end of the experiment. The maximum percentage of germinated seed (plateau) was reached between the second and third day with a mean germination rate spanning from 78.8±5.09% (CPT) to 87.77±6.94% (DMSO_C) **(Supplementary Figure 3A**). Similarly, the germination speed (T_50_) was not affected by the treatments as most seeds germinated within one to two days (**Supplementary Figure 3B**). The high standard deviations bars generally indicate non-uniform seed germination. These results suggest that treatments with the indicated concentrations of DMSO and CPT do not affect germination percentage and speed.


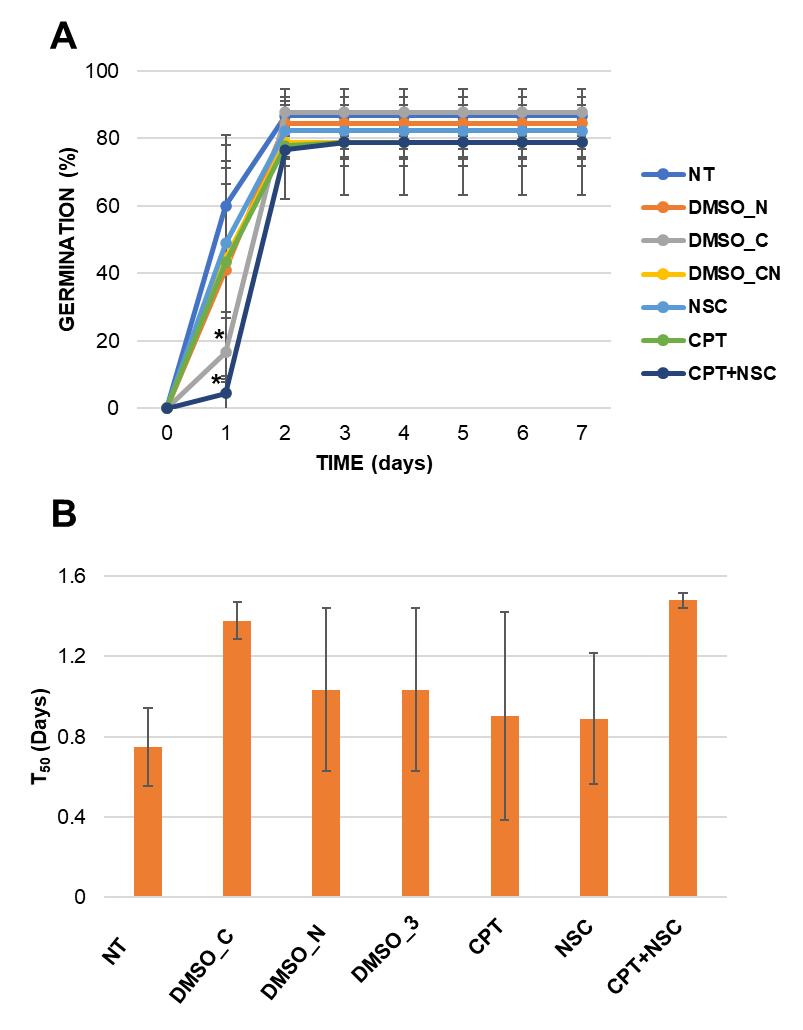


**Supplementary Figure 3**. Evaluation of the effect of imposed CPT, NSC, and CPT+NSC treatments and corresponding DMSO concentrations (DMSO_N, DMSO_C, DMSO_CN) on *Medicago truncatula* seed germination. (**A**) Germination percentage (%). (**B**) Germination speed, T_50_ (days). Data are represented as mean ± standard deviation of three independent replicates. Statistically significant (*P*<0.05) differences between treatments and control (NT) are represented with an asterisk (*). CPT, camptothecin; NSC, TDP1 inhibitor NSC120686; DMSO, dimethyl sulfoxide; NT, non-treated control.

- 1. **The effect of DMSO treatments on gene expression profiles**

From the previous analyses we observed that the used DMSO concentrations to dissolve the CPT/NSC agents did not influence seedling growth. However, it was necessary to also test if DMSO could have an influence on the expression of the selected genes. It is thus possible to observe changes in the expression profiles of some genes in the DMSO samples compared to the NT control. For example, *TDP1α* seems to be upregulated by DMSO_C and DMSO_CN, while topoisomerase genes seem to be downregulated by the DMSO treatments (**Supplementary Figure 4A**). In the case of other DNA repair genes, *MUS81* appears to be mostly affected by the DMSO_N treatment (**Supplementary Figure 4B**). In the case of the genes involved in cell cycle regulation, cyclin genes also seem to be influenced mostly by DMSO_N and DMSO_CN (**Supplementary Figure 4C**).


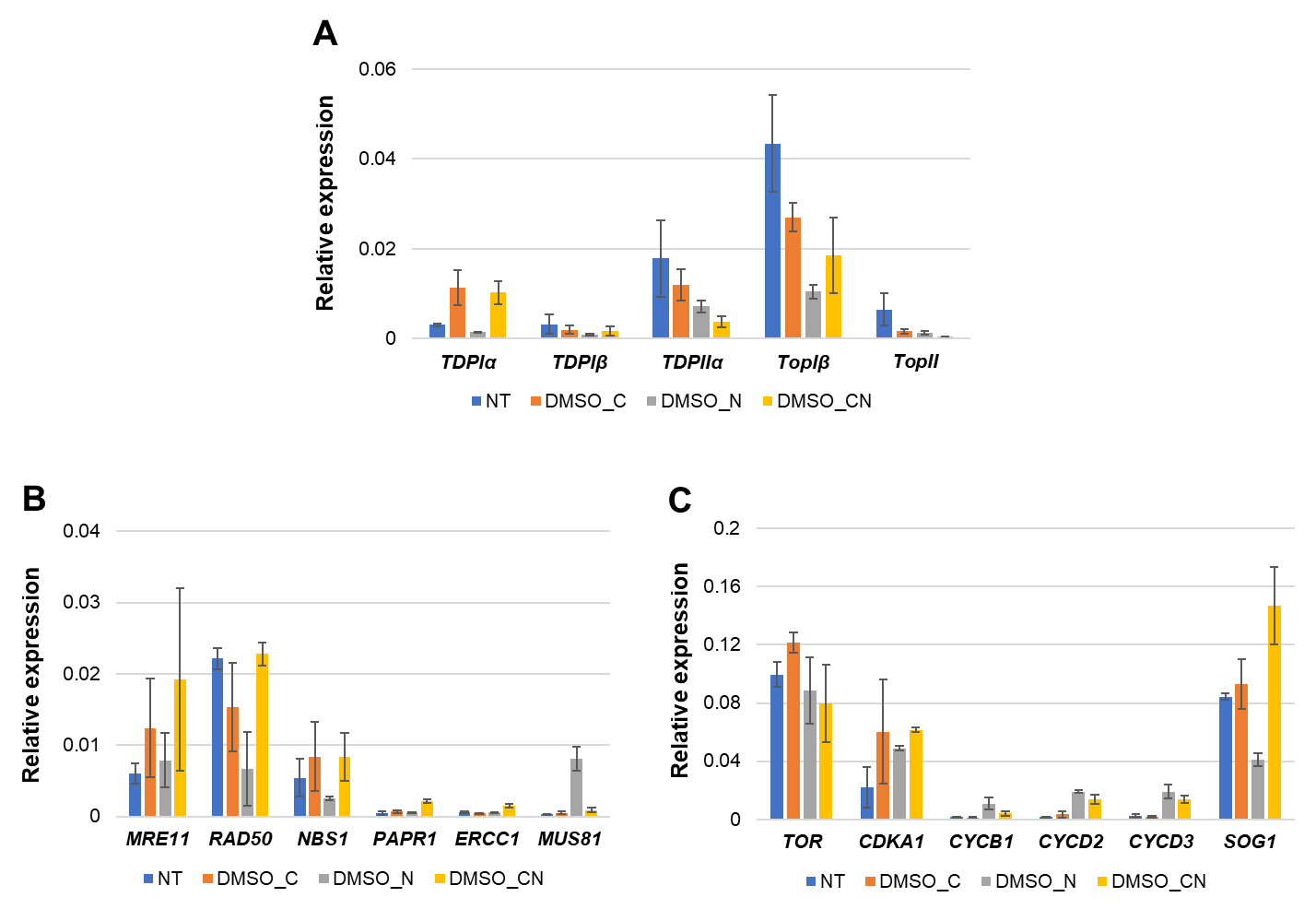


**Supplementary Figure 4**. Expression profiles in non-treated (NT) and DMSO-treated samples at different concentrations. (**A**) Tyrosyl-DNA phosphodiesterases and topoisomerases genes. (**B**) Genes involved in TDP1-alternative repair pathways. (**C**) Genes involved in cell cycle regulation. Data are represented as mean ± standard deviation of technical replicates.

**Supplementary Table 1**. Oligonucleotide sequences used for the amplification of genes through qRT-PCR reactions.

| GENE | ACCESION NO. | FORWARD PRIMER (5’-3’) | REVERSE PRIMER (3’-5’) |
| --- | --- | --- | --- |
| REFERENCE GENES | | | |
| *ELF1α* | Medtr6g021805 | GACAAGCGTGTGATCGAG | TTTCACGCTCAGCCTTAA |
| *ACT* | Medtr3g095530 | TCAATGTGCCTGCCATGTATG | ACTCACACCGTCACCAGAATC |
| *Tub* | Medtr7g089120 | TTTGCTCCTCTTACATCCCGTG | GCAGCACACATCATGTTTTTGG |
| *Ubi* | Medtr3g091400 | GCAGATAGACACGCTGGGA | AACTCTTGGGCAGGCAATAA |
| *PDF2* | Medtr6g084690 | GTGTTTTGCTTCCGCCGTT | CCAAATCTTGCTCCCTCATC |
| *PPRrep* | Medtr6g079830 | GGAAAACTGGAGGATGCACG | CAAGCCCTCGACACAAAACC |
| *GAPDH* | Medtr3g085850 | TGCCTACCGTCGATGTTTCAGT | TTGCCCTCTGATTCCTCCTTG |
| GENES OF INTEREST | | | |
| *TOR* | Medtr5g005380 | TGATGTTACCGTACGCCACT | TAAAGCGGCAAATACTGCAC |
| *TopIα* | Medtr0172s0010 | ATACACGTGGGCTATTGTCG | TCACTTGGATGAATGCGTT |
| *TopII* | Medtr3g031040 | AGGATCCGTCGTGGGATTGTAAGGC | ACAACAGAGAGGCCAGCCATAG |
| *TDP1α* | Medtr7g050860 | ACGAGTTGGGAGTGCTCTTT | GGGATTTATCCTTCGATTGTTT |
| *TDP2* | Medtr4g132300 | CAGATGTTCAGCAAGGAACG | CCCGTCTTGCAAAGGATATT |
| *TDP1β* | Medtr8g095490 | GGTTGGTTTGAGCCATCTTT | GCAGGCACATTGTGATTTCT |
| *PARP1* | Medtr1g088375 | AAACCCACCCTCCTTCGT | GTCCCTCGGTCTCTTTCC |
| *NBS1* | *Medtr5g076180* | *TGCAAACCCGATTTCAATAA* | *GATGAAATAAGCACGCATGG* |
| *RAD50* | Medtr3g084300 | GGCGAGAAAGTTGTTTGCCTTAG | GCCAATTTGCTTCATCTTGA |
| *ERCC1* | Medtr1g082570 | CGTTCGTCAAATCCTCAGAA | TGAAGCTGCAGGAGCATTAT |
| *MRE11* | Medtr2g081100 | TCCAAAGTGGTGCTGATGA | ATGGATTCATTGTCCGAACTG |
| *MUS81* | Medtr3g022850 | AAGAAGCCACTGGATTGTTCC | ATTTGGATGGCTTCTGGAAA |
| *CDKA1* | Medtr4g094430 | CGTCTTGAGCAGGAAGAT | TCCTGTGCTGCATTTCTT |
| *CycD2* | Medtr5g032550 | GGCTCTTGATTGGATTT | ACAAGTCACACCTTCTGGA |
| *CycB1* | Medtr5g088980 | AACTCATGGCGAGCTTTC | AGCAACAGCACAACGATC |
| *CycD3* | Medtr3g102310 | ACAGCGTTGAGCCTAGTTTAG | TTCATACCCTGACCACAG |
| *ACYLTR* | Medtr2g089765 | CGCCTCTTGATCTTCCTTCAC | GAATCTCGAACCAAACCCGC |
| *AGO1A* | Medtr6g477980 | TGACAGTGGCTCAATGACAA | GGGGTCTAACAGCAGCATTA |
| *ATUBC2* | Medtr4g108080 | TACGATGTTGCTGCGATTCT | TCACGCTTGTTCTCACTGAA |
| *E2FE-like* | Medtr4g106540 | CAGGCGCCTTTATGATATTGC | AGCCACCTGAATGCTGGTTT |
| *DMAP1* | Medtr1g086590 | TGCATGCTTTCGTTAGGTGG | AGTTGAGTTCACTGCTGCTT |
| *RAD54-like* | Medtr5g004720 | CGTTGCCAAAACAATGATGGG | AGCCTGCAATCTCAGCAAATC |
| *SOG1* | Medtr5g053430 | TGGTGCGAAGGGACAGATAA | TCACACAAGGACAATGCGTC |

**Supplementary Table 2**. Two-tailed primers used for the retro-transcription (RT) reaction. Accession numbers, relative to miRbase database (<http://www.mirbase.org/>) are indicated.

| miRNA | ACCESION NO. | RT PRIMER (5’-3’) |
| --- | --- | --- |
| mtr-miR156a | MIMAT0001654 | TCTTCTGTCAGCTTGAGTCCTCGTAGAGTTGCTACGAGATATGATAATGTGCT |
| mtr-miR168a | MIMAT0011089 | CACCAAGCAACAACGACCAGAGCTAGAGAACCTAGCTCACCCACTACTTCCCG |
| mtr-miR172c-5p | MIMAT0021265 | ATGATGCTACCGACGAATACTGCTAGAGTTGCTAGCAGAGCCCTTAATGTGAA |
| mtr-miR2600e | MIMAT0021331 | CACAATGCTTCAACGACCAGAGCTAGAGAACCTAGCTCACCCACTACGCCAAT |
| mtr-miR395e | MIMAT0003858 | ACACTTCATCAACGACCAGAGCTAGAGAACCTAGCTCACCCACTACGAGTTC |
| mtr-miR5741a | MIMAT0023118 | TTAGTCCCTATCAAGCTCTCCAGGTACAGTTGGTACCTGACTCCACGCAAACCA |

Supplementary Table 3. Oligonucleotide sequences used for the amplification of microRNAs through qRT-PCR reaction.

| miRNA | ACCESION NO. | FORWARD PRIMER (5’-3’) | REVERSE PRIMER (3’-5’) |
| --- | --- | --- | --- |
| mtr-miR156a | MIMAT0001654 | CGATGCTACCGACGAATACTG | GCCATCATCATCAAGATTCACA |
| mtr-miR168a | MIMAT0011089 | GCCACCAAGCAACAACGAC | GATGGTGCTGGTCGGGAA |
| mtr-miR172c-5p | MIMAT0021265 | ATGATGCTACCGACGAATACTG | GTAGCATCATCAAGATTCACA |
| mtr-miR2600e | MIMAT0021331 | CACAATGCTTCAACGACCAGAG | AAGCATTGTGGCATTGTGATTGGC |
| mtr-miR395e | MIMAT0003858 | ACACTTCATCAACGACCAGAG | ATGAAGTGTTTGGGGGAACTC |
| mtr-miR5741a | MIMAT0023118 | TTAGTCCCTATCAAGCTCTCCAG | TAGGGACTAAATTGATGGTTT |

**Supplementary Table S4.** Output of the one way ANOVA on ranks performed with Kruskal-Wallis test on variables including the expression profiles of all investigated genes and miRNAs. Chi-square values, degrees of freedom (df) and *p*-values are indicated. *P*-values < 0.05 are considered as significant.

| **Variable** | **Kruskal-Wallis**  **chi-square** | **df** | ***p*-value** |
| --- | --- | --- | --- |
| *SOG1* | 16.3188255613126 | 5 | 0.00599037890524766 |
| *TOR* | 7.54970760233918 | 5 | 0.182860555558371 |
| *TDP1α* | 12.3471216821786 | 5 | 0.0303288648795763 |
| *TDP1β* | 10.3312650810065 | 5 | 0.0663750199731925 |
| *TDP2α* | 13.6832126852809 | 5 | 0.0177519809005963 |
| *Top1β* | 15.8348909657321 | 5 | 0.00733162332745209 |
| *Top2* | 14.7309941520468 | 5 | 0.0115757761241677 |
| *Rad50* | 14.5204678362573 | 5 | 0.0126203892885176 |
| *MRE11* | 9.14035087719299 | 5 | 0.103595291548881 |
| *NBS1* | 14.6140350877193 | 5 | 0.0121453117491813 |
| *PARP1* | 14.9803719008264 | 5 | 0.0104465357912321 |
| *ERCC1* | 13.6739130434783 | 5 | 0.0178189776571118 |
| *MUS81* | 13.8654970760234 | 5 | 0.0164867515786378 |
| *Cdka1* | 5.1744226128921 | 5 | 0.394966595649292 |
| *Cycb1* | 15.8020725388601 | 5 | 0.0074324829135712 |
| *Cycd2* | 12.9800068941744 | 5 | 0.0235668472183489 |
| *Cycd3* | 15.2690058479532 | 5 | 0.0092726827839593 |
| *AGO1* | 13.0134297520661 | 5 | 0.0232532413083834 |
| *UBE2A* | 15.6198830409357 | 5 | 0.00801742938738133 |
| *5AT* | 16.5789473684211 | 5 | 0.0053715966757529 |
| *E2FE* | 14.8479532163743 | 5 | 0.0110321554118708 |
| *DMAP1* | 16.7169614984391 | 5 | 0.00506907788643818 |
| *RAD54-like* | 8.36842105263158 | 5 | 0.137066118267334 |
| mtr.miR168a | 15.7225094794898 | 5 | 0.00768263802187958 |
| mtr.miR156a | 16.215564738292 | 5 | 0.00625483151388716 |
| mtr.miR2600e | 16.6670164393145 | 5 | 0.00517658358254548 |
| mtr.miR172-5p | 15.1911157024793 | 5 | 0.00957619424265617 |
| mtr.miR395e | 15.2229145032883 | 5 | 0.0094511375394545 |
